# Synergistic Effects of Diabetes, Inflammatory/Coagulopathy Biomarkers, TG and Hypoxic Stress on Premature Coronary Heart Disease in High-Altitude Populations

**DOI:** 10.3390/jcdd13080372

**Published:** 2026-08-06

**Authors:** Jianbo Yu, Danzeng Quzhen, Luosang Quzhen, Peiliang Gao, Jue’a Ciren, Xueying Fu, Jihong Zhu, Zhenzhong Yang, Guiying Dong

**Affiliations:** 1 Emergency Department, Xizang Autonomous Region People’s Hospital, Lhasa 850000, China; yujianbo@sina.cn (J.Y.); 15289197806@163.com (D.Q.); lsqz@163.com (L.Q.); jueazeren@126.com (J.C.); 2 Emergency Department, Peking University People’s Hospital, Beijing 100044, China; gaopeiliang@pkuph.edu.cn (P.G.); fuxueying@pkuph.edu.cn (X.F.); zhujihong642021@163.com (J.Z.)

**Keywords:** acute coronary syndrome, premature, inflammation, coagulatory, diabetes mellitus

## Abstract

**Background:** Risk profiles of premature coronary heart disease (PCHD, ≤45 years) among high-altitude acute coronary syndrome (ACS) populations remain poorly characterized. This study explored clinical features and independent predictors of early-onset ACS in Lhasa. **Methods:** We retrospectively enrolled 467 first-ACS patients (1 July 2022–30 June 2024). Patients were further dichotomized into PCHD and non-PCHD groups for comparison. Univariable and multivariable logistic regression identified PCHD risk factors. **Results:** Compared with non-PCHD patients, PCHD patients featured higher proportions of male non-Tibetans, higher smoking and alcohol consumption rates, lower prevalence of hypertension and diabetes, and elevated SpO_2_ (all *p* < 0.05). They had significantly higher CHOL, TG, LDL-C, UA and Hb (all *p* < 0.05), accompanied by lower WBC, PLT and D-dimer (all *p* < 0.001). Multivariate regression confirmed diabetes mellitus (OR = 1.158, *p* = 0.014), TG (OR = 1.625, *p* < 0.001), WBC (OR = 1.995, *p* = 0.007), D-dimer (OR = 2.841, *p* = 0.043) and SpO_2_ (OR = 0.937, *p* = 0.019) as independent predictors of PCHD. **Conclusions:** Integrated monitoring of glucose, lipids, hypoxia and thromboinflammatory biomarkers may supply preliminary auxiliary reference for early prevention of PCHD in local highland youth, while large multi-center verification is still needed to confirm its clinical value.

## 1. Introduction

With the acceleration of population aging and urbanization, cardiovascular diseases (CVDs) have an increasingly significant impact on health, and the incidence of CVDs continues to increase. Among the five major health problems announced by the WHO, the incidence of coronary heart disease (CHD) ranks the second in male disease burden and the third in female disease burden [1,2]. It is estimated that the current number of CHD patients in China is about 11.39 million, of which about 3% occur under the age of 40. Without intervention, China will face 6.1 million acute myocardial infarction (AMI)-related hospital admissions annually by 2030 [3], and there will be a trend towards younger age groups. Research data from China shows that the proportion of young and middle-aged AMI patients is about 56.5% [4], and the incidence rate among young people under 45 years old is as high as 7% to 10% [5].

Premature CHD (PCHD, onset < 45 years) portends significantly amplified long-term cardiovascular risk, with 63.7% of patients experiencing recurrent ischemic events and death within 5 years, even if they receive timely coronary revascularization and secondary prevention treatment [6]. PCHD’s potential high economic burden makes it an important clinical problem [7,8]. Therefore, identifying the characteristics and risk factors of acute coronary syndrome (ACS) in early-aged people, and timely prevention and treatment of PCHD, are of great significance for delaying or improving prognosis [9,10].

While Global Burden of Disease meta-regressions focus on plain multi-regional cardiovascular cohorts [11], our research targets a special population residing at altitudes over 3600 m in Lhasa. Long-term continuous high-altitude hypoxic stimulation acts as an exclusive environmental risk factor that cannot be ignored in plateau residents, which is fundamentally different from plain populations without chronic hypoxic exposure. This unique environmental stimulus independently triggers compensatory erythrocytosis, vascular endothelial damage, lipid metabolic disorders and inflammatory–coagulopathy imbalance, and synergistically accelerates the onset of premature coronary heart disease (PCHD). Compared with ordinary ACS studies based on lowland crowds, altitude hypoxia is the core distinctive feature of our study population and the core pathogenic background of this cohort, which endows our data with irreplaceable research value for plateau cardiovascular diseases [12]. In this work, we have sought to improve understanding of ACS morbidity and mortality in adult patients of different ages in plateau populations, especially young adults, with the goal of verifying the mediating effect of clinical factors on confirmed PCHD in Xizangan populations, thereby bridging the systematic research gap in this demographic.

## 2. Materials and Methods

### 2.1. Population and Data Source

Patient data were sourced from the Xizang Autonomous Region Chest Pain Quality Control Center Registry for all emergency department-presenting admissions between 1 July 2022 and 30 June 2024. Hospitalization metrics were extracted from institutional electronic healthcare record systems.

**Figure 1:**
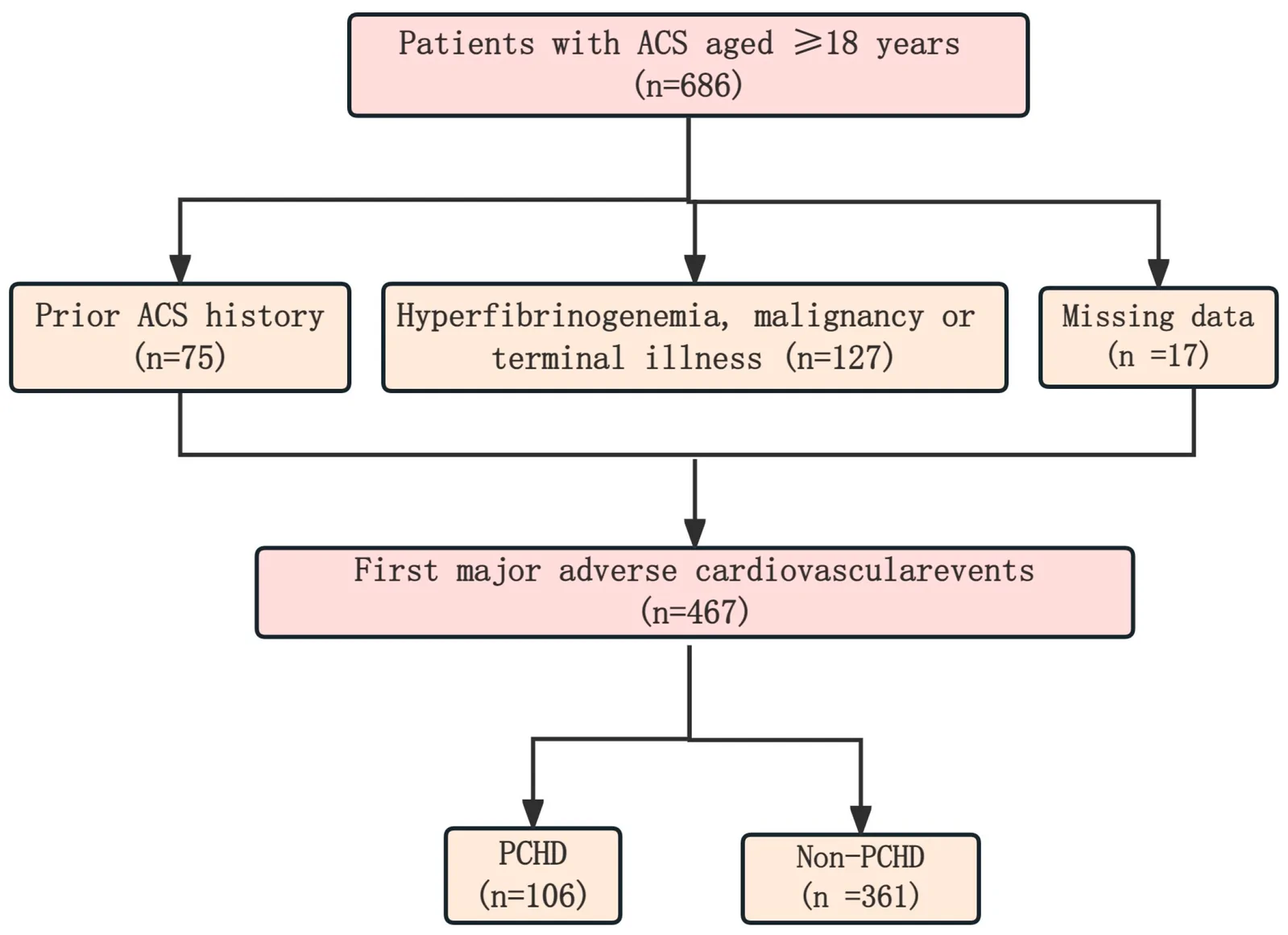
Flow diagram of patient selection.

We enrolled patients aged ≥18 years with ACS whose diagnosis complied with the 2023 European Society of Cardiology Clinical Practice Guidelines [13]. Exclusion criteria were as follows: (1) prior ACS history at admission (*n* = 75); (2) baseline hyperfibrinogenemia, malignancy or terminal illness (*n* = 127); (3) incomplete clinical data (*n* = 17); (4) pregnancy or puerperium (*n* = 0). After screening, 467 eligible patients aged 22–90 years were retained for final analysis. According to age at ACS onset, all participants were dichotomized into PCHD (≤45 years) and non-PCHD (>45 years) groups. The patient selection flowchart is shown in Figure 1.

Emergency laboratory tests covered routine hematology, coagulation and biochemical panels. Fasting venous blood sampled on hospital day 2 was assayed for lipid profiles and homocysteine (Hcy). Complete blood counts were detected with a Sysmex XS-1000i hematology analyzer (Sysmex, Kobe, Japan); serum biochemistry was measured on an Abbott ARCHITECT c16000 platform (Abbott, Chicago, IL, USA). Coagulation indices were quantified via clot waveform analysis using a STA MAX coagulation analyzer (Stago, Gennevilliers, France). Recorded biomarkers included white blood cell (WBC), platelet (PLT), hemoglobin (Hb), fibrinogen (FIB), D-dimer, uric acid (UA), total cholesterol (CHOL), triglycerides (TG), high-density lipoprotein cholesterol (HDL-C), low-density lipoprotein cholesterol (LDL-C) and Hcy.

Notably, all peripheral oxygen saturation (SpO_2_) values were measured on room air at the first emergency admission before any supplemental oxygen was administered, avoiding interference from oxygen therapy on saturation readings.

### 2.2. Statistical Analysis

Statistical analyses were conducted using SPSS 25.0 (SPSS Inc., Chicago, IL, USA). Normality of variable distributions was verified via the Kolmogorov–Smirnov test. Non-normally distributed continuous data, expressed as median (interquartile range, IQR), were compared with the Mann–Whitney U test. Categorical variables, presented as counts (percentages), were analyzed using the χ^2^ test or Fisher’s exact test as appropriate. Univariate and multivariate logistic regression models were constructed to identify independent risk factors for PCHD. A two-tailed *p* < 0.05 indicated statistical significance.

## 3. Results

### 3.1. Demographics and Clinical Characteristics

This Lhasa-based retrospective observational study enrolled 467 patients with first-onset ACS, consisting of 410 males and 57 females. We dichotomized all participants into PCHD (*n* = 106) and non-PCHD (*n* = 361) groups for targeted comparison, with full demographic, clinical and laboratory characteristics summarized in Table 1.

Among all enrolled patients, 8 (1.7%) died during hospitalization; 49 (10.5%) chose to receive palliative care at home or transferred to other hospitals due to personal wishes; and 410 (87.8%) were discharged with clinical improvement. Baseline comparisons stratified by PCHD status revealed distinct intergroup differences (Table 1). Briefly, the PCHD group featured a higher proportion of males (98.1% vs. 84.8%; *p* < 0.001) and non-Tibetan residents (42.5% vs. 69.5%; *p* < 0.001), along with higher prevalence of current smoking (66.0% vs. 51.2%; *p* = 0.007) and alcohol drinking (79.2% vs. 63.4%; *p* = 0.002). PCHD patients had significantly higher peripheral oxygen saturation (SpO_2_, 92 vs. 90%; *p* = 0.002) relative to non-PCHD counterparts. In terms of comorbidities, hypertension (34.9% vs. 48.2%; *p* = 0.016) and diabetes mellitus (2.8% vs. 11.6%; *p* = 0.007) were less common in PCHD patients.

AMI was more frequent as the index admission diagnosis in PCHD patients (92.5% vs. 84.2%, *p* = 0.031). No in-hospital deaths were recorded in the PCHD subgroup, while 16.0% of young ACS patients discharged against medical advice.

### 3.2. Laboratory Biomarkers

Compared with non-PCHD patients, PCHD individuals had significantly elevated median CHOL (4.35 vs. 4.02 mmol/L, *p* = 0.001), TG (1.54 vs. 1.42 mmol/L, *p* < 0.001), LDL-C (2.66 vs. 2.50 g/L, *p* = 0.014) and Hb (173 vs. 160 g/L, *p* < 0.001). No significant difference in HDL-C (0.86 vs. 0.87 g/L, *p* = 0.062) and Hcy (15.42 vs. 16.24 μmol/L, *p* = 0.795) was observed. Median UA was slightly higher in the PCHD group (390 vs. 384 μmol/L, *p* = 0.004).

PCHD patients exhibited higher median WBC (7.1 vs. 6.4 × 10^9^/L, *p* < 0.001) and D-dimer concentration (0.29 vs. 0.22 mg/L, *p* < 0.001). Median PLT count was also higher in the PCHD group (220 vs. 211 × 10^9^/L, *p* < 0.001), while FIB levels were similar between subgroups (3.1 vs. 2.4 g/L, *p* = 0.397).

### 3.3. Risk Factors for PCHD

Multicollinearity diagnostic tests were performed for all candidate covariates prior to regression modeling, with full tolerance and variance inflation factor (VIF) outputs summarized in Table S1. Only CHOL and LDL-C presented severe multicollinearity (VIF > 20), while all remaining predictors exhibited VIF values below 2, indicating no substantial collinear interference for subsequent regression analysis.

Univariate and multivariate logistic regression were applied to identify predictors of PCHD (Table 2). Unadjusted analysis revealed significant correlations between PCHD and sex, ethnicity, smoking, alcohol consumption, hypertension, diabetes, SpO_2_, UA, WBC, PLT and D-dimer (all *p* < 0.05), whereas CHOL and LDL-C showed no relevance.

After comprehensive adjustment, SpO_2_ [OR 0.937 (95% CI, 0.887–0.939), *p* = 0.019] diabetes mellitus [OR 1.158 (95% CI, 1.038–1.654), *p* = 0.014], TG [OR 1.625 (95% CI, 1.482–1.809), *p* = 0.001], WBC [OR 1.995 (95% CI, 1.992–1.999), *p* = 0.007] and D-dimer [OR 2.841 (95% CI, 1.035–7.809), *p* = 0.043] emerged as independent risk factors for PCHD.

## 4. Discussion

Although the overall incidence of atherosclerotic cardiovascular disease has declined over the past two decades [14,15], the prevalence of cardiovascular disease among young adults aged 15–39 years continues to rise worldwide [11]. Existing registry data confirm that males bear a higher risk of premature CHD; a Portuguese cohort including 29,870 ACS patients demonstrated that 25% of participants presented early-onset coronary lesions with a mean onset age of 50 ± 7 years, and STEMI was the dominant clinical phenotype [16]. Approximately 85% of in-hospital deaths related to ACS occur in patients aged ≥65 years [17]. Endogenous estrogen produced in premenopausal women exerts multi-target cardioprotective effects, including ameliorating vascular endothelial function, inhibiting lipid peroxidation, reducing the secretion of inflammatory cytokines and suppressing platelet aggregation, thereby markedly lowering the risk of formation and rupture of early atherosclerotic plaques [18]. This well-documented estrogen-mediated protective mechanism largely accounts for the extremely low proportion of female patients (only 1.9%) diagnosed with premature coronary heart disease in this plateau cohort.

We compared demographic, clinical and laboratory indicators between premature CHD (PCHD, onset ≤ 45 years) and non-PCHD patients admitted to Lhasa hospital. Young early-onset ACS cases were mostly migrant non-Tibetan males with higher smoking and alcohol intake rates, whereas local native Tibetans constituted the majority of elderly ACS patients. Heavy clustering of tobacco and alcohol exposure among young males represents a distinctive lifestyle feature of plateau PCHD, consistent with previous domestic epidemiological studies focusing on young cardiovascular populations [19]. Young patients also had lower rates of hypertension and diabetes. Laboratory testing demonstrated significantly elevated TG, CHOL, LDL-C and Hb in patients with PCHD, while median levels of white blood cell, platelet and D-dimer were higher relative to the non-PCHD group. Univariate analysis detected significant intergroup differences in gender, ethnicity, smoking, alcohol consumption, UA and PLT, yet these variables lost statistical significance after full multivariate adjustment. Univariate and multivariate logistic regression were performed to screen independent predictors of PCHD, and five independent predictors were identified: SpO_2_, diabetes mellitus, TG, WBC and D-dimer.

This finding aligns with previous cohort studies verifying that the triglyceride–glucose (TyG) index can predict atherosclerotic cardiovascular and cerebrovascular risks [20,21,22]. Diabetes exacerbates endothelial dysfunction and acts as the most powerful comorbid risk factor for young patients with ACS. A previous registry reported that 92% of ACS patients carried at least one conventional cardiovascular risk factor, and 67% presented risk factor clustering. Classic cardiovascular hazards including diabetes, hypercholesterolemia, smoking and hypertension collectively advance coronary heart disease onset by roughly 10 years in vulnerable populations [23]. Chronic altitude hypoxia synergistically amplifies vascular endothelial injury induced by hyperglycemia, while triglyceride-rich lipoproteins accelerate plaque deposition and instability under sustained hypoxic stimulation. Notably, univariate crude comparison showed lower diabetes prevalence in the PCHD subgroup. After adjusting ethnicity, lifestyle and lipid confounders, diabetes independently increases the risk of premature ACS. The inconsistent trend of diabetes arose from strong age confounding in crude intergroup comparison. Elderly patients accumulate higher rates of chronic metabolic diseases over time, leading to higher raw diabetes prevalence in the non-PCHD cohort. After full covariate adjustment, diabetes exerts independent pro-atherogenic effects on young plateau populations. Hyperglycemia amplifies hypoxic endothelial damage, oxidative stress and thromboinflammation under high-altitude environments, which explains why diabetes acts as an independent predictor of premature coronary heart disease despite its low overall proportion in young ACS patients.

Although WBC and D-dimer emerged as independent risk factors in regression models, thromboinflammatory perturbations merely serve as synergistic auxiliary mediators under unique high-altitude conditions, rather than primary pathogenic drivers. ACS stems from a self-amplifying loop of plaque inflammation and thrombosis. Lipid plaque inflammation induces macrophage MMP-9 and neutrophil NET release, triggering plaque rupture; exposed tissue factor and adherent platelets then activate extrinsic coagulation to form occlusive coronary thrombi [24,25]. Elevated inflammatory markers predict CHD severity and prognosis [15]. M1 macrophages, NET-releasing neutrophils and Th1 lymphocytes mediate immune–thrombotic crosstalk, rendering inflammation both a biomarker and pathogenic driver of atherosclerosis [26]. IL-6 upregulates fibrinogen under inflammation, an effect amplified by RAAS activation; fibrinogen further boosts IL-6 to sustain proinflammatory feedback. This RAAS–thrombosis–inflammation triad preferentially destabilizes plaques in young adults [27,28]. Despite well-defined thromboinflammatory CHD pathways, relevant quantitative data in young angina/AMI patients remain scarce. Our highland cohort fills this gap and reveals the unique pathophysiology of plateau PCHD.

Long-term hypoxic exposure triggers prominent metabolic [29,30] and hematological disturbances among young plateau residents [31], which collectively initiate and accelerate early coronary atherosclerotic lesions. Distinct adaptive erythropoiesis exists between native Tibetans and young migrant populations, which partly explains intergroup differences in hemoglobin and thrombotic risk [32]. Indigenous Tibetans form stable hypoxic acclimatization with moderately elevated Hb without excessive blood viscosity [33,34]. By contrast, young migrants lack long-term plateau adaptation; their reactive polycythemia markedly raises whole blood viscosity, slows microcirculatory perfusion, and increases the risk of local intracoronary thrombosis [35,36]. This hematological maladaptation synergizes with hypertriglyceridemia and hyperglycemia to amplify early coronary lesion risk in migrant youth [37].

As a unique plateau-derived biomarker, higher SpO_2_ exerted protective effects against early coronary events, proving that sustained hypoxia impairs vascular endothelial function and accelerates atherosclerosis progression. Mechanistically, hypoxia drives ischemia–reperfusion injury, sterile inflammation and thrombosis [38,39]. Chronic high-altitude exposure raises IL-1β, IL-6 and TNF-α; excess reactive oxygen species and proinflammatory cytokines induced by hypoxia jointly damage endothelium and accelerate atherosclerosis [40].

Based on the stratified phenotypic characteristics of PCHD and non-PCHD patients, targeted screening strategies can be formulated for plateau young residents. For ACS patients aged ≤45 years, routine monitoring of TG, SpO_2_ and hemoglobin should be prioritized, while standardized intervention for smoking and alcohol cessation is essential to cut off the upstream mediating pathways of lipid and erythrocyte abnormalities. For elderly ACS patients, regular surveillance of hypertension, diabetes and thromboinflammatory markers (WBC, D-dimer) should be emphasized due to accumulated age-related inflammatory burden.

This study has several limitations worthy of attention. First and foremost, this study is a retrospective single-center observational study with a limited sample size. Retrospective data collection inevitably introduces unmeasured baseline confounders and potential selection bias, which may affect the reliability and generalizability of our statistical results. Although all participants are local residents with consistent high-altitude living backgrounds and constitute a unique plateau ACS cohort unavailable in plain-area studies, the small sample restricted the extrapolation of our findings to different highland regions. Second, we failed to complete long-term follow-up for patients who discharged against medical advice. Local cultural customs make many high-risk patients choose palliative care at home, which may lead to underestimation of true cardiovascular mortality in young ACS patients and distort the in-hospital mortality result we observed. Third, we observed no in-hospital deaths among patients with premature coronary heart disease (PCHD) during their admission. Nevertheless, nearly 16.0% of young ACS patients requested discharge against medical advice; many of these patients opted to seek palliative care or transfer to inland medical facilities, and their long-term survival status could not be tracked due to unavailable follow-up data. As a result, the actual long-term cardiovascular mortality risk in young patients may be underestimated. Fourth, the severe gender imbalance (98.1% male in the PCHD group) restricts the applicability of our findings to female young ACS patients. Fifth, only admission cross-sectional biomarkers were collected, without long-term follow-up data to evaluate the predictive efficiency of these indicators on long-term cardiovascular adverse events.

To overcome the biases originating from retrospective single-center data, we have formulated a prospective multi-center research plan covering multiple altitude zones and multi-ethnic plateau populations. Standardized unified data collection protocols will be adopted to reduce unmeasured confounders and minimize selection bias in follow-up validation research. Prospective multi-center cohort studies covering multiple altitudes and ethnic groups are needed to further validate the mediating mechanism of lipids and hemoglobin in PCHD. We plan to expand sample size by enrolling participants from medical centers with different altitude gradients, so as to quantitatively verify the synergistic pathogenic interaction between high-altitude chronic hypoxia and glycolipid, inflammatory and coagulation biomarkers on premature coronary lesions. Stratified risk prediction models separately for young and elderly plateau ACS patients should be constructed to realize age-specific risk stratification. In addition, more precise inflammatory indicators such as high-sensitivity CRP and glycated hemoglobin can be incorporated to further clarify the synergistic interaction of hypoxia, glycolipid metabolism and thrombosis in the pathogenesis of premature coronary heart disease.

## 5. Conclusions

This study identified independent predictive biomarkers, including diabetes, triglycerides, inflammatory and coagulation indicators, as well as the hypoxic marker SpO_2_, associated with premature coronary heart disease among Lhasa high-altitude young residents. Young plateau ACS patients are characterized by prominent lipid disorders but mild baseline thromboinflammation. Nevertheless, due to the retrospective single-center design, limited sample size and absence of long-term post-discharge follow-up, these biomarkers only provide preliminary regional observational clues rather than mature, widely applicable clinical screening indicators. Large-scale multi-center prospective cohorts covering diverse altitudes and ethnic groups are required to further verify their clinical predictive value. Routine combined detection of blood glucose, lipid, inflammatory, coagulation and blood oxygen indicators can supply auxiliary risk reference for local young plateau residents to prevent early-onset coronary lesions.

## Figures and Tables

**Table 1:** Patient demographics and clinical characteristics.

	All (*n* = 467)	PCHD (*n* = 106)	Non-PCHD (*n* = 361)	*p* Value
Male, *n* (%)	410 (87.8%)	104 (98.1%)	306 (84.8%)	<0.001 *
Tibetan, *n* (%)	296 (63.4%)	45 (42.5%)	251 (69.5%)	<0.001 *
Altitude, median (IQR), m	3657 (3652, 4005)	3657 (3561, 3657)	3657 (3653, 4023)	0.110
BMI, median (IQR), kg/m^2^	25.1 (22.6, 28.0)	24.7 (22.4, 29.2)	25.4 (22.7, 27.7)	0.690
Family history of CVD, *n* (%)	72 (15.4%)	21 (19.8%)	51 (14.1%)	0.154
Alcohol drinking, *n* (%)	255 (54.6%)	70 (66.0%)	185 (51.2%)	0.007 *
Current smoker, *n* (%)	313 (67.0%)	84 (79.2%)	229 (63.4%)	0.002 *
**Metabolic biomarker**				
CHOL, median (IQR), mmol/L	3.99 (3.38, 4.73)	4.35 (3.64, 4.90)	4.02 (3.54, 4.71)	0.001 *
TG, median (IQR), mmol/L	1.31 (1.02, 1.76)	1.54 (1.18, 2.57)	1.42 (1.09, 1.88)	<0.001 *
HDL-C, median (IQR), g/L	0.91 (0.77, 1.06)	0.86 (0.75, 1.02)	0.87 (0.76, 1.03)	0.062
LDL-C, median (IQR), g/L	2.45 (1.86, 3.08)	2.66 (2.17, 3.00)	2.50 (2.04, 3.14)	0.014 *
Hcy, median (IQR), μmol/L	16.28 (12.65, 22.64)	15.42 (12.36, 26.72)	16.24 (12.43, 20.69)	0.795
UA, median (IQR), μmol/L	397 (340, 475)	390 (349, 532)	384 (336, 450)	0.004 *
Hb, median (IQR), g/L	166 (150, 183)	173 (164, 190)	160 (150, 181)	<0.001 *
**Inflammation and coagulation biomarker**				
WBC, median (IQR), *10^9^/L	8.7 (6.6, 11.95)	7.1 (5.4, 9.0)	6.4 (5.5, 7.7)	<0.001 *
FIB, median (IQR), g/L	3.1 (1.4, 4.0)	3.1 (1.1, 4.0)	2.4 (1.3, 4.0)	0.397
PLT, median (IQR), *10^9^/L	214 (176, 264)	220 (157, 264)	211 (169, 237)	<0.001 *
D-dimer, median (IQR), mg/L	0.30 (0.22, 0.56)	0.29 (0.22, 0.46)	0.22 (0.19, 0.31)	<0.001 *
**Measurements**				
SBP, median (IQR), mmHg	120 (106, 136)	112 (103, 120)	124 (106, 139)	0.218
DBP, median (IQR), mmHg	77 (68, 88)	72 (66, 84)	78 (68, 90)	0.718
SPO_2_, median (IQR), %	90 (88, 93)	92 (90, 93)	90 (87, 93)	0.002 *
**Medical history**				
Hypertension, *n* (%)	211 (45.2%)	37 (34.9%)	174 (48.2%)	0.016 *
Diabetes mellitus, *n* (%)	45 (9.6%)	3 (2.8%)	42 (11.6%)	0.007 *
Dyslipidemia, *n* (%)	20 (4.3%)	4 (3.8%)	16 (4.4%)	0.087
**Admission event**				
AMI, *n* (%)	402 (86.1%)	98 (92.5%)	304 (84.2%)	0.031 *
**Clinical outcomes**				
Hospitalization death, *n* (%)	8 (1.7%)	0 (0.0%)	8 (2.2%)	0.208 ^F^
Against medical advice, *n* (%)	49 (10.5%)	17 (16.0%)	32 (8.9%)	0.034 *
Rehabilitation, *n* (%)	410 (87.8%)	89 (84.0%)	321 (88.9%)	0.170

Data are presented as the median (IQR) or *n* (%). * *p* < 0.05 was considered statistically significant. ^F^, Fisher’s exact test. AMI, acute myocardial infarction. BMI, body mass index. DBP, diastolic blood pressure. SBP, systolic blood pressure. CHOL, cholesterol. TG, triglycerides. LDL-C, low-density lipoprotein cholesterol. HDL-C, high-density lipoprotein cholesterol. WBC, white blood cell. Hb, hemoglobin. PLT, platelet. Hcy, homocysteine. UA, uric acid. FIB, fibrinogen.

**Table 2:** Association between clinical risk factors and the first incident PCHD.

Variables	Univariable OR (95% CI)	*p* Value	Multivariable OR (95% CI)	*p* Value
Male	0.107 (0.026, 0.446)	0.002 *		
Tibetan	0.311 (0.199, 0.486)	<0.001 *		
Current smoker	2.201 (1.314, 3.687)	0.003 *		
Alcohol drinking	1.850 (1.177, 2.907)	0.008 *		
SPO_2_	0.935 (0.893, 0.979)	0.004 *	0.937 (0.887, 0.989)	0.019 *
Hypertension	0.576 (0.368, 0.903)	0.016 *		
Diabetes mellitus	1.221 (1.067, 1.729)	0.013 *	1.158 (1.038, 1.654)	0.014 *
TG	1.518 (1.404, 1.666)	<0.001 *	1.625 (1.482, 1.809)	<0.001 *
UA	0.997 (0.995, 0.999)	0.002 *		
Hb	0.851 (0.802, 0.903)	<0.001 *		
WBC	1.986 (1.978, 1.994)	0.001 *	1.995 (1.992, 1.999)	0.007 *
PLT	0.995 (0.992, 0.998)	<0.001 *		
D-dimer	7.99 (2.956, 21.597)	<0.001 *	2.841 (1.035, 7.809)	0.043 *

* *p* < 0.05 was considered statistically significant. CHOL, total cholesterol; LDL-C, low-density lipoprotein cholesterol; TG, triglycerides; WBC, white blood cell count; Hb, hemoglobin; PLT, platelet count; UA, uric acid; SpO_2_, peripheral oxygen saturation.

## Data Availability

The original contributions presented in this study are included in the article/supplementary material. Further inquiries can be directed to the corresponding authors.

## Supplementary Materials

The following supporting information can be downloaded at https://www.mdpi.com/article/10.3390/jcdd13080372/s1. Table S1: Collinearity diagnosis of all candidate covariates for multivariate logistic regression of PCHD.

## Abbreviations

The following abbreviations are used in this manuscript:

PCHD	premature coronary heart disease
ACS	acute coronary syndrome
CVD	cardiovascular diseases
AMI	acute myocardial infarction
Hcy	homocysteine
WBC	white blood cell
PLT	platelet
Hb	hemoglobin
FIB	fibrinogen
UA	uric acid
CHOL	cholesterol
TG	triglycerides
HDL-C	high-density lipoprotein cholesterol
LDL-C	low-density lipoprotein cholesterol
