# Supplementary material for: Synergistic Effects of Diabetes, Inflammatory/Coagulopathy Biomarkers, TG and Hypoxic Stress on Premature Coronary Heart Disease in High-Altitude Populations
Source: J Cardiovasc Dev Dis. 2026 Aug 6;13(8):372. doi: 10.3390/jcdd13080372 (PMC13513838; doi:10.3390/jcdd13080372)
Supplement: Supplementary file 1 [file jcdd-13-00372-s001.zip › jcdd-4453169-supplementary.pdf]

**Table S1. Collinearity diagnosis of all candidate covariates for multivariate logistic regression of PCHD.**

| Model | Covariate | Unstandardized coefficient (B) | Standard error | Standardized coefficient (Beta) | t-value | *P*-value | Tolerance | VIF |
|---|---|---|---|---|---|---|---|---|
| 1 | (Constant) | 2.742 | 0.405 | — | 6.766 | 0.000 | — | — |
| | Gender | 0.086 | 0.066 | 0.067 | 1.294 | 0.196 | 0.674 | 1.485 |
| | Tibetan ethnicity | 0.069 | 0.042 | 0.081 | 1.648 | 0.100 | 0.752 | 1.330 |
| | Current smoking | 0.011 | 0.048 | 0.013 | 0.239 | 0.811 | 0.642 | 1.559 |
| | Alcohol drinking | -0.021 | 0.043 | -0.025 | -0.493 | 0.622 | 0.705 | 1.419 |
| | $SpO_2$ | -0.006 | 0.003 | -0.076 | -1.706 | 0.089 | 0.909 | 1.100 |
| | Hypertension | 0.058 | 0.037 | 0.068 | 1.543 | 0.124 | 0.939 | 1.065 |
| | Diabetes mellitus | 0.172 | 0.062 | 0.121 | 2.760 | 0.006 | 0.953 | 1.049 |
| | CHOL | -0.035 | 0.059 | -0.120 | -0.585 | 0.559 | 0.044 | 22.969 |
| | TG | -0.073 | 0.025 | -0.166 | -2.888 | 0.004 | 0.553 | 1.808 |
| | LDL-C | 0.033 | 0.057 | 0.117 | 0.587 | 0.557 | 0.046 | 21.674 |
| | UA | 0.000 | 0.000 | -0.035 | -0.741 | 0.459 | 0.833 | 1.200 |
| | Hb | -0.001 | 0.001 | -0.071 | -1.423 | 0.155 | 0.723 | 1.382 |
| | WBC | -0.012 | 0.006 | -0.111 | -2.206 | 0.028 | 0.725 | 1.379 |
| | PLT | -0.001 | 0.000 | -0.113 | -2.400 | 0.017 | 0.816 | 1.225 |
| | D-dimer | 0.103 | 0.056 | 0.085 | 1.825 | 0.069 | 0.846 | 1.182 |

Tolerance < 0.1 or variance inflation factor (VIF) > 10 indicates severe multicollinearity.

CHOL, total cholesterol; LDL-C, low-density lipoprotein cholesterol; TG, triglycerides; WBC, white blood cell count; Hb, hemoglobin; PLT, platelet count; UA, uric acid; $SpO_2$, peripheral oxygen saturation. Dependent variable: PCHD status (1 = PCHD, 0 = non-PCHD).
